# Redox Regulation of Nonmuscle Myosin Heavy Chain during Integrin Engagement

**DOI:** 10.1155/2012/754964

**Published:** 2011-12-19

**Authors:** Tania Fiaschi, Giacomo Cozzi, Paola Chiarugi

**Affiliations:** Department of Biochemical Sciences, University of Florence, Viale Morgagni 50, 50134 Florence, Italy

## Abstract

On the basis of our findings reporting that cell adhesion induces the generation of reactive oxygen species (ROS) after integrin engagement, we were interested in identifying redox-regulated proteins during this process. Mass spectrometry analysis led us to identify nonmuscle myosin heavy chain (nmMHC) as a target of ROS. Our results show that, while nmMHC is reduced in detached/rounded cells, it turns towards an oxidized state in adherent/spread cells due to the integrin-engaged ROS machinery. The functional role of nmMHC redox regulation is suggested by the redox sensitivity of its association with actin, suggesting a role of nmMHC oxidation in cytoskeleton movement. Analysis of muscle MHC (mMHC) redox state during muscle differentiation, a process linked to a great and stable decrease of ROS content, shows that the protein does not undergo a redox control. Hence, we propose that the redox regulation of MHC in nonprofessional muscle cells is mandatory for actin binding during dynamic cytoskeleton rearrangement, but it is dispensable for static and highly organized cytoskeletal contractile architecture in differentiating myotubes.

## 1. Introduction

Studies over the past years have shown that reactive oxygen species (ROS) are involved in a diverse array of biological processes, including normal cell growth, induction and maintenance of cell transformation, programmed cell death, and cellular senescence. ROS are able to trigger such divergent responses probably through differences in the level and duration of the oxidant burst or in the cellular context accompanying oxidative stress. ROS include a variety of partially reduced oxygen metabolites (e.g., superoxide anions, hydrogen peroxide, and hydroxyl radicals) with a higher reactivity with respect to molecular oxygen. Oxidants can either be produced within cells by dysfunction of mitochondrial respiratory chain complexes or by cytosolic or membrane recruited enzymes such as NADPH oxidase, cyclooxygenases, lipoxygenases, and the NO synthase [1].

 Oxidants have been proposed as intracellular messengers of a variety of physiological stimuli acting on cytosolic oxidases. Considerable progress has been made in identifying intracellular targets of ROS. Several findings support the idea that exogenous ROS or oxidants produced by activation of growth factors receptors or integrins can reversibly oxidize and hence inactivate redox-sensitive proteins. Proteins with low-p*K*_*a*_ cysteine residues, which are vulnerable to oxidation by hydrogen peroxide_,_ include several transcription factors, such as the nuclear factor *κ*-B [2], activator protein 1 [3], hypoxia-inducible factor [4], p53 [5], the p21Ras family of proto-oncogenes [6], phosphotyrosine phosphatases (PTPs) [7], and src kinase family [8]. Our findings provide evidence that intracellular ROS are generated following integrin engagement and that these oxidant intermediates are necessary for integrin signalling during fibroblasts adhesion and spreading [9]. ROS production in response to integrin engagement represents signalling integration point between extracellular matrix (ECM) and growth factor signalling, and they are produced in Rac1 and 5- lipoxygenase- (5-LOX-) dependent manner [9]. A key role in the cytoskeleton redox regulation is played by a low molecular weight-phosphotyrosine phosphatase (LMW-PTP), which is oxidised/inhibited in response to ECM contact. Its inactivation prevents the dephosphorylation of two key regulators of cytoskeleton dynamics: the focal adhesion kinase (FAK) [9] and a GTPase activating protein for the GTPase RhoA (p190RhoGAP) [10]. Accordingly, the redox-dependent activation of FAK and p190RhoGAP leads to focal adhesion formation, membrane ruffles development, and cell spreading [9, 11, 12]. Hence, both the small GTPases Rac1 and RhoA are critical regulators of redox-mediated actin cytoskeleton remodelling during cell spreading and migration.

Significantly, the key role of ROS in integrin signalling suggests that they may contribute to malignant growth and invasiveness through a deregulation of cell/matrix interaction and cell motility. We are now interested in identifying, among cytoskeleton proteins, the molecular targets of ROS in anchorage-dependent growth. To date, specific targets of integrin-generated ROS are LMW-PTP and SH2-PTP [9, 13], the tyrosine kinase Src [8, 9] and actin [9, 14, 15]. Oxidation of these proteins produces differential effects: (i) LMW-PTP and SH-PTP2 are inactivated through formation of an intramolecular disulfide [9, 13]; (ii) Src family kinases are conversely activated through a disulfide which blocks the protein in active state [8]; (iii) *β*-actin is oxidized through glutathionylation in a single sensitive cysteine, thus leading to increased polymerization and stress fiber formation [15].

We report herein that nonmuscle myosin heavy chain (nmMHC) is oxidized in the early stage of integrin-mediated adhesion in human fibroblasts. This redox control retains a functional role during cytoskeleton dynamic rearrangements in response to ECM contact, strongly affecting nmMHC binding of *β*-actin. Conversely, during stable and static cytoskeletal organization in contractile myotubes, the association of muscle MHC (mMHC) with actin is redox independent, suggesting a selective role of redox regulation of these proteins only during dynamic rearrangements of cytoskeleton.

## 2. Materials and Methods

### 2.1. Materials

Unless specified, all reagents were obtained from Sigma-Aldrich. Human fibroblasts were obtained as already described [16]. Anti-pan-actin and antimuscle Myosin Heavy Chain antibodies were from Santa Cruz Biotechnology, Nonmuscle myosin heavy chain antibodies were from Biomedical Technologies Inc. Anti-GSH antibodies were obtained from Virogen. N-(biotinoyl)-N′-(iodoacetyl)ethylene diamine (BIAM) was obtained from Molecular Probes. The streptavidin-horseradish (Strp-HRP) conjugate was from Biorad. Immunopure immobilized streptavidin was from Pierce Biotechnology. Blotting grade blocker nonfat dry milk was from Biorad. Secondary antibodies were from Amersham Bioscience. Sequencing grade modified trypsin was from Promega.

### 2.2. Cell Culture

Human fibroblasts were obtained as previously described [17]. Human fibroblasts and murine myoblasts C2C12 were cultured in Dulbecco's modified Eagle's medium (DMEM) supplemented with 10% calf serum at 37°C in a 5% CO_2_ humidified atmosphere. To induce myogenic differentiation, C2C12 cells were grown until subconfluence and then cultured for six days in differentiating medium containing DMEM supplemented with 2% horse serum.

### 2.3. Cell Adhesion Assay

Human fibroblasts were serum deprived for 24 h and then detached with 0.25% trypsin for 1′. Trypsin digestion was then blocked by the use of 0.5 mg/mL soybean trypsin inhibitor. Cells were then centrifuged and diluted in fresh culture media and incubated for 30 minutes in gentle agitation at 37°C. The adherent/spread sample was obtained plated the cells on fibronectin-coated dishes for 45 minutes while the detached/cell rounded sample was plated for the same time on polylysine-coated plates. Nordihydroguaiaretic acid (NDGA) was added to the cells at the beginning of the suspension phase at a final concentration of 10 *μ*M.

### 2.4. Intracellular H_2_O_2_ Assay

For the measure of ROS generation during adhesion, cells were serum deprived for 24 h, detached and incubated in gentle agitation for 30 minutes. Cells were then plated on fibronectin-coated dishes in serum-free medium, with or without NDGA 10 *μ*M, and ROS assay was performed at different times of adhesion. Three minutes before the end, 5 *μ*M DCF-DA was added. Cells were lysed in 1 mL of RIPA buffer containing 1% Triton X-100 and fluorescence was analysed immediately using a Perkin Elmer Fluorescence Spectrophotometer (excitation wavelength 488 nm, emission wavelength 510 nm). The values of fluorescence were normalized on the proteins content. The assay of ROS production in myoblasts and in six days differentiating myotubes were performed using the same protocol.

### 2.5. In Vivo BIAM Labelling of Proteins

Cells from adherent/spread and detached/rounded conditions are lysates in RIPA buffer (50 mM Tris-HCl, pH 7,5, 150 mM NaCl, 1% Triton, 2 mM EGTA) supplemented with BIAM (100 *μ*M final concentration) and protease inhibitors cocktail (1 mM AEBSF, 8 *μ*M aprotinin, 20 *μ*M leupeptin, 40 *μ*M bestatin, 15 *μ*M pepstatin A, and 14 *μ*M E-64). Lysates were then maintained on ice for 15 minutes and then centrifuge at 13000 rpm for 15′. For the binding of BIAM-labelled proteins with immobilized streptavidin, 30 *μ*L of resin were added to the clarified samples and maintained overnight at 4°C in gentle agitation [12]. The resin was firstly washed four times with RIPA buffer and then resuspended in Laemmli sample buffer. The pattern of BIAM-labelled proteins were visualized by a Western blot analysis using horseradish peroxidase-streptavidin conjugate, washed and developed with the enhanced chemiluminescence kit.

### 2.6. Matrix Assisted Laser Desorption Ionization-Time of Light (MALDI-TOF) Sample Preparation

BIAM-labelled lysates from spread and rounded cells were run on SDS-PAGE. The gel was then stained by Coomassie blue solution, subjected to destaining solution for 24 h, and finally washed in water until completely equilibrated. The bands of interest were excised, transferred to an Eppendorf tube, and then washed twice with 50 mM NH_4_HCO_3_/acetonitrile (1 : 1), and they were shrunk with acetonitrile. After drying, samples were subjected to a reduction reaction in a buffer containing 10 mM dithiothreitol, 25 mM NH_4_HCO_3_ for 45 minutes at 56°C followed by an alkylation step in a buffer containing 55 mM Iodoacetic acid, 25 mM NH_4_HCO_3_ for 30 minutes at room temperature in the dark. After a final washing step, samples were dried up and trypsin digested for 24 h at 37°C. The peptides were then extracted from gel bands by sonification and by supplementing 50% acetonitrile and 1% trifluoroacetic acid (1 : 1 proportion with sample), and the supernatants were recovered and then dried.

### 2.7. MALDI-TOF Analysis

Spectrometric analysis were conducted on an Ultraflex MALDI-TOF (Bruker Daltonics) using a Scout ion source and operating in positive reflector mode. Samples were mixed with a-Cyano-4-hydroxycinnamic acid (1 : 1). 0.8 pmol/uL of sample were deposed with the dry droplet technique on an AnchorChip target. Peptides were identified within an error of 120 part per million. Mascot search algorithm parameters was set as following: carboxylation of cysteine and oxidation of methionine.

### 2.8. Immunoprecipitation and Western Blot Analysis

Immunoprecipitation was performed overnight using 2 *μ*g/mL of specific antibodies. Immunocomplexes were collected on protein A-Sepharose, separated by SDS-poly-acrylamide gel electrophoresis, and transferred onto PVDF membrane. Immunoblots were probed firstly with specific antibodies in 2% nonfat dry milk, 0,05% Tween 20 in phosphate buffered saline buffer, and then with secondary antibodies conjugated with horseradish peroxidase, washed, and developed with the enhanced chemiluminescence kit.

### 2.9. Statistical Analysis

Data are presented as means ± S.D from at least three experiments. Analysis of densitometry was performed using Quantity One Software (Bio-Rad). Statistical analysis of the data was performed by Student's *t*-test. *P* values ≤0.05 were considered statistically significant.

## 3. Results and Discussion

### 3.1. Redox Regulation of Nonmuscle MHC during Cell Adhesion

**Figure 1:**
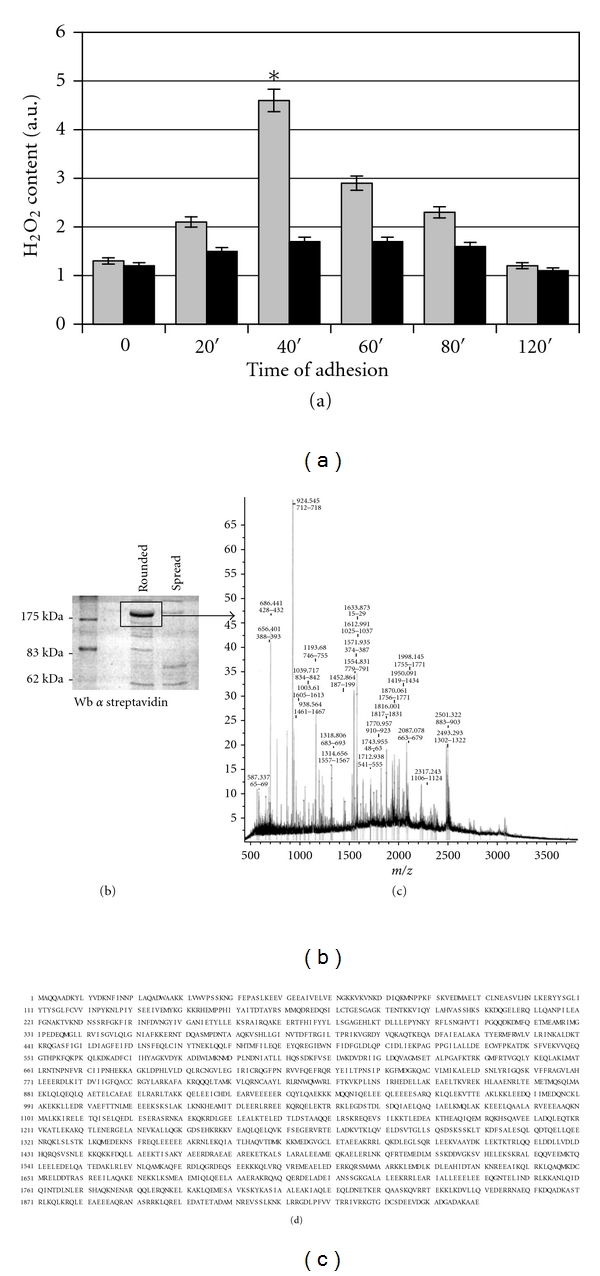
Identification of nmMHC as a redox-sensitive protein during cell adhesion. (a) Content of H_2_O_2_ in human fibroblasts during adhesion on fibronectin. Hydrogen peroxide production was assayed used DCF-DA. *P < 0.001 versus time 0. (b) Cell lysates from rounded and spread human fibroblasts were labelled with BIAM and then added to immobilized streptavidin. The pattern of proteins were visualized by a treatment with HRP conjugate streptavidin. (c) Nonredundant (nrNCBI) database was scanned using MASCOT search algorithm. The nonmuscle myosin heavy chain IX of mus musculus (gi/20137006) was identified with a significant score of 115 at 120 ppm mass tolerance. The spectrum shows the matched peaks. (d) The primary structure of nmMHC is shown.

Firstly, we found that the engagement of integrin during cell adhesion induces in human fibroblasts a transient burst of ROS production (with a peak 40 minutes after fibronectin attachment) in keeping with what observed during spreading and adhesion of NIH-3T3 murine fibroblasts [9]. ROS burst was significantly inhibited by the use of NDGA which affects the activity of 5-LOX, thus confirming 5-LOX as the source of ROS production (Figure 1(a)) [9]. To study redox-regulated proteins during integrin-mediated cell adhesion, we used the BIAM-labelling technique. BIAM is a sulfhydryl-modifying reagent that selectively probes the thiolate form of cysteine residues, as already reported by Kim et al. [18]. Human fibroblasts were serum deprived for 24 h, detached, and maintained in suspension for 30 minutes to eliminate integrin signalling. For adherent/spread conditions, cells were left to adhere for 45 minutes on fibronectin-coated plates, while detached/rounded cells were seeded on polylysine-coated dishes. The analysis of redox-regulated proteins during integrin engagement shows the presence of a major band of about 200 KDa, differently labelled with BIAM in rounded and spread cells, suggesting a redox regulation of this protein during cell adhesion (Figure 1(b)). We repeated the experiment described above, performing a Coomassie staining of the SDS-PAGE gel, and the BIAM labelled band was excised and used for MALDI-TOF analysis. After trypsin digestion, the peptide fragments were probed on a MALDI-TOF mass spectrometer, and the fingerprint data were then submitted to the Mascot search algorithm. Figure 1(c) shows the analysis of the spectrum of the digested peptides that identify the protein as nonmuscle myosin heavy chain (nmMHC) with a score of 107 (Figure 1(d)).

### 3.2. The Redox State of nmMHC Affects *β*-Actin Association

**Figure 2:**
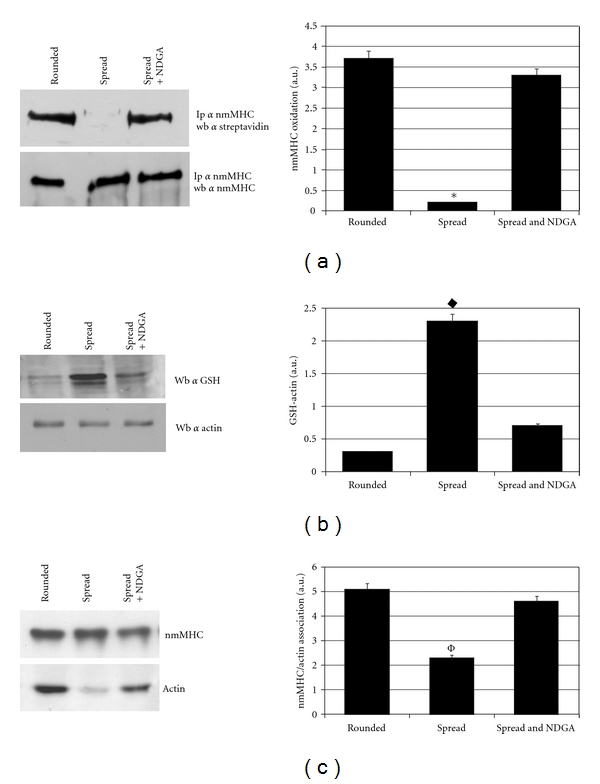
Analysis of nmMHC redox state and *β*-actin association in human fibroblasts. Cells lysates from detached and spread cells, treated with or without NDGA, were labelled with BIAM and nmMHC was immunoprecipitated with specific antibodies. (a) BIAM labelled pattern of nmMHC was revealed by western blot using HRP-streptavidin conjugate; amount of immunoprecipitated nmMHC was obtained probing the membrane with anti-nmMHC antibodies; the histogram shows the ratio between two corresponding samples after densitometric evaluation. *P < 0.001 versus rounded. (b) Representative immunoblot showing actin glutathionylation in rounded and spread human fibroblasts. The histogram corresponds to the ratio between GSH-actin and total actin. ^♦^ *P* < 0,001 versus rounded. (c) Analysis of nmMHC-actin association. An anti-nmMHC immunoprecipitation was performed from rounded and spread cells treated with or without NDGA. The detection of actin associated with nmMHC was obtained with antiactin immunoblot, while anti-nmMHC immunoblot was used for normalization. The histogram corresponds to the ratio between the values obtained by densitometric analysis of two corresponding samples of the blots. ^Φ^ *P* < 0.005 versus rounded. Similar results were obtained in four independent experiments. a.u.: arbitrary units.

Firstly, we confirmed the data obtained by MALDI-TOF analysis by nmMHC immunoprecipitation. Results indicated that nmMHC is reduced in rounded cells and turns towards the oxidized form in spread cells. Furthermore, the treatment of the cells with NDGA, which abrogates ROS production by 5-LOX, rescues the reduced form of nmMHC (Figure 2(a)). In the same experimental setting, we found a redox regulation of *β*-actin, which became oxidised in spread cells through the binding with glutathione (Figure 2(b)).

 Myosin is the main motor protein of the cell and carries on this function through its binding with *β*-actin. The role of this association is the generation of the force responsible for cellular dynamic functions such as locomotion, cell division, and cytoplasmic contraction [19]. Therefore, we investigated whether the nmMHC oxidation, upon complete cell spreading, influences its association with *β*-actin. Results clearly show a redox sensitivity of the binding of nmMHC to *β*-actin. Indeed, treatment of spread fibroblasts with the 5-LOX inhibitor NDGA rescues the lack of binding between *β*-actin and nmMHC observed upon completion of the spreading process (Figure 2(c)).

In addition to other redox-regulated proteins of cytoskeleton, such as actin and profilin [15, 20, 21], our data add nmMHC as a new cytoskeletal protein undergoing redox regulation during spreading of human fibroblasts.

### 3.3. Redox State and mMHC/Actin Association in Differentiating Myotubes

**Figure 3:**
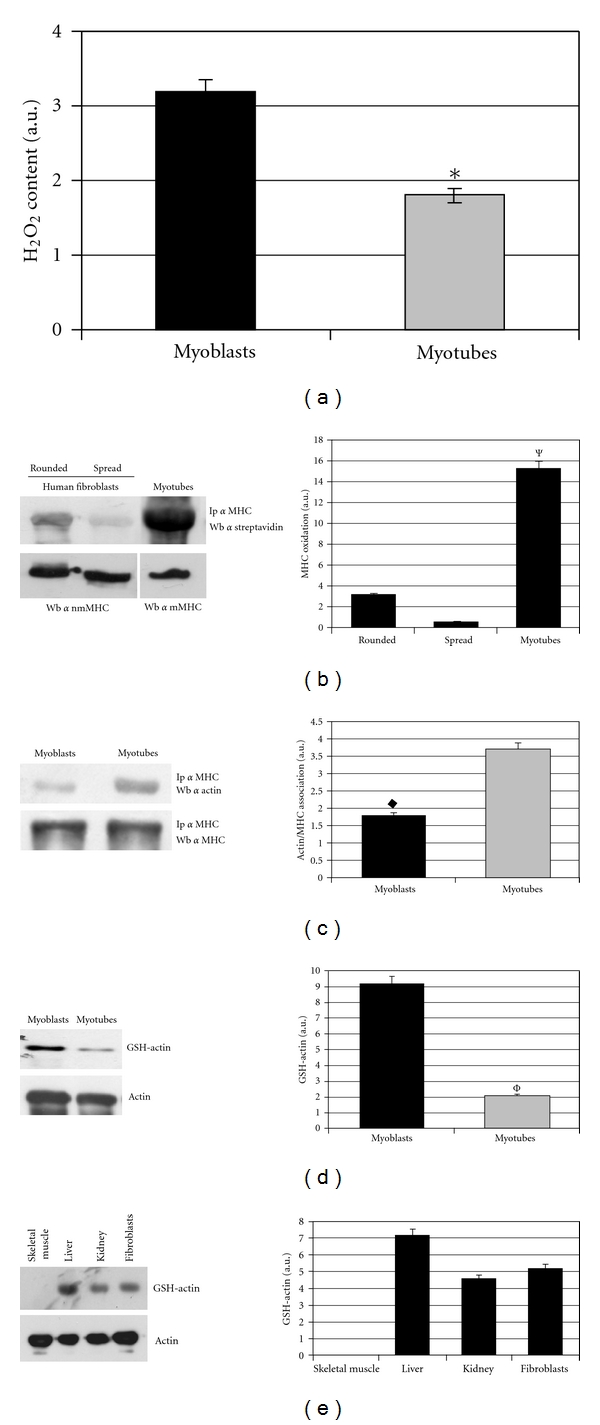
Analysis of the redox state of muscle MHC and actin in differentiating myotubes. (a) Analysis of H_2_O_2_ content in myoblasts and in differentiating myotubes after six days of differentiation. Hydrogen peroxide assay was performed with DCF-DA. *P < 0.005 versus myoblasts. (b) Analysis of the redox state of MHC on rounded or spread human fibroblasts and in differentiating myotubes. BIAM labelled pattern of MHC was revealed by a Western blot using HRP-streptavidin conjugate after immunoprecipitation of nmMHC in human fibroblasts and mMHC in differentiating myotubes; MHC normalization was performed probing the membrane with specific anti-MHC antibodies; the histogram corresponds to the ratio between two corresponding values obtained by densitometric analysis. ^Ψ^ *P* < 0.001. (c) Analysis of MHC-actin association in myoblasts and in differentiating myotubes. An anti-nmMHC immunoprecipitation was performed from myoblasts and from differentiating myotubes. The amount of actin associated with MHC was obtained with antiactin immunoblot, while the normalization was performed using anti-MHC antibodies. The histogram reports the ratio between the values obtained by densitometric analysis of the two corresponding values of the blots. ^♦^ *P* < 0.0015 versus myotubes. (d) and (e) Analysis of actin glutathionylation. Actin glutathionylation was assayed on growing C2C12 myoblasts, differentiating myotubes, human fibroblasts, and in murine skeletal muscle, liver, and kidney using anti-GSH antibodies. ^Φ^ *P* < 0.001 versus myotubes. a.u.: arbitrary units.

In agreement with previous results [22], we observed that differentiation of murine myoblasts C2C12 is associated with a decrease of ROS content (Figure 3(a)). On the basis of these results, we speculated that muscle MHC (mMHC) might not be redox regulated in resting conditions, that is, when cytoskeleton does not undergo rearrangements due to the movement or spreading onto ECM. Therefore, we compared MHC redox state in spread or rounded fibroblasts and in differentiating myotubes. The results clearly showed that MHC in differentiating myotubes is mainly in its reduced form, while in spread fibroblasts MHC undergoes redox control (Figure 3(b)).

It is well known that muscle differentiation is accompanied with a dramatic and stable rearrangement of cytoskeleton architecture accompanied with the formation of contractile fibers. It is likely that binding of myosin to actin behaves as a cyclic event, and it is not under redox control. As expected, the results show that MHC/actin association is greatly improved in differentiating myotubes with respect to myoblasts (Figure 3(c)). Again, when ROS are high (as in spread fibroblasts or in undifferentiated myoblasts), *β*-actin is not bound to MHC. Conversely, when ROS are low (as in rounded/detached fibroblasts or in differentiating myotubes), actin strictly binds MHC, thereby underscoring the key importance of the redox control of the two proteins for their regulated interaction.

We have previously demonstrated that the ROS burst produced by ECM-integrin binding induces oxidation of *β*-actin, through glutathionylation of cysteine 374, which is an essential step for actomyosin disassembly [15]. Whether glutathionylation is important for its binding to myosin, we expected that in differentiating myotubes, where the association between these two proteins is essential for contraction, actin should be less glutathionylated. As expected, we found that actin glutathionylation is greater in myoblasts with respect to differentiating myotubes, in agreement with their ROS content in differentiated cells (Figure 3(d)). Furthermore, analysis of several murine tissues reveals that skeletal muscle fibres contains reduced actin, while in liver, kidney, and skin fibroblasts *β*-actin is oxidised/glutathionylated (Figure 3(e)). This suggests that both actin and MHC in skeletal muscle must be reduced to allow their continuous association for muscle contraction.

Although acute production of ROS have positive effects in skeletal muscle (such as for glucose uptake), ROS accumulation can provoke serious consequences for skeletal muscle physiology [23]. Beyond serious skeletal muscle pathologies (such as Duchenne muscular dystrophy and mitochondrial myopathies), in which an increased/uncontrolled production of ROS has been reported [24], oxidative molecules alter the physiology also in healthy muscle. Indeed, high amount of ROS induce an acute decrease in force production during repeated contractions, accompanied with a lower Ca^2+^ sensitivity of myofibrils [25]. In addition, several studies reported slower fatigue development in the presence of ROS scavengers [26]. In agreement with these observations and with our results, a decreased muscle contraction in oxidative conditions has been reported, suggesting that molecules involved in these process should not meet oxidation [27, 28].

**Figure 4:**
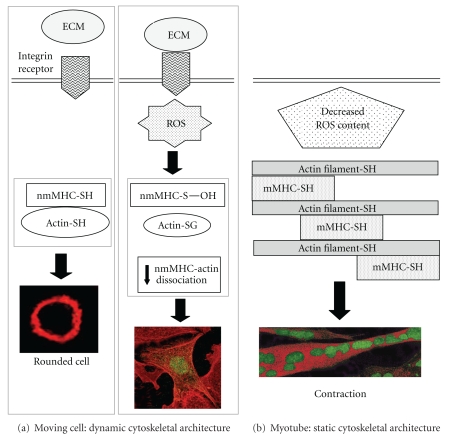
Proposed model for the MHC oxidation during fibroblasts adhesion and muscle differentiation. In a rounded cell, where level of ROS are low, both nmMHC and actin are reduced and associated ((a), left). In the early stage of fibroblast adhesion, the engagement of integrin receptors generates a burst of ROS leading to oxidation of nmMHC and decreased its binding with actin ((a), right). It is likely that the redox-dependent nmMHC/actin association is functional to the cytoskeleton dynamics during cell motility. Myotubes, where a stable and static cytoskeletal structure has been formed, are characterized by a low ROS content. In this environment, nmMHC is not redox-regulated and the increased nmMHC/actin association is a redox-independent mechanism that guarantees contraction (b).

The different behaviour of MHC in growing fibroblasts and in differentiating myotubes could be explained considering the function and architecture of cytoskeleton in these two situations. In undifferentiated growing cells, cytoskeleton is a dynamic structure which is subjected to continuous remodelling in response to extracellular signals to allow cell-shape changes associated with directed movement, secretion, or cell division (Figure 4(a)). Conversely, muscle differentiation is associated to a great cytoskeletal rearrangements that culminate with the formation of a static structure. In this view, it is possible that the actin/MHC binding does not need any further (i.e., transient) regulatory mechanism, as they are already associated to form stable structures (Figure 4(b)). On the contrary, in nonmuscle cells this association may be finely regulated, since both *β*-actin and MHC can be rapidly assembled and disassembled in response to extracellular signals. This control may be obtained through ROS generated by integrins during cell adhesion, where oxidants might act as second messengers for actin/MHC cytoskeleton remodelling (Figure 4).

Taken together, these findings demonstrate that also proteins forming the cytoskeletal architecture could be ROS-regulated and that this control might be important for the cytoskeleton rearrangement during some cellular functions. Furthermore, these results open new perspectives for future investigations, such as the evaluation of ROS sensitivity of cytoskeletal proteins in cancer cells, a phenomenon linked both to a great ROS increase and a strong cytoskeleton rearrangements.

## Abbreviations

BIAM: N-(biotinoyl)-N′-(iodoacetyl)ethylene diamine
FAK: Focal adhesion kinase
HRP: Horse radish peroxidase
GSH: Glutathione
LMW-PTP: Low molecular weight-phosphotyrosine phosphatase
5-LOX: 5-lipoxygenase
MHC: Myosin heavy chain
mMHC: muscle myosin heavy chain
nmMHC: Nonmuscle MHC
NDGA: Nordihydroguaiareticacid
ROS: Reactive oxygen species.
